# Performance Analysis of Underlay Cognitive Radio System with Self-Sustainable Relay and Statistical CSI

**DOI:** 10.3390/s21113727

**Published:** 2021-05-27

**Authors:** Nadica Kozić, Vesna Blagojević, Predrag Ivaniš

**Affiliations:** School of Electrical Engineering, University of Belgrade, 11000 Belgrade, Serbia; vesna.golubovic@etf.rs (V.B.); predrag.ivanis@etf.rs (P.I.)

**Keywords:** channel state information, cognitive radio, cooperative relay network, decode-and-forward, energy harvesting, Rayleigh fading, outage probability

## Abstract

The relentlessly increasing number of small-sized devices with limited powering and computational capabilities requires the adoption of new approaches to spectrum access. In this paper, we analyze an underlay cooperative cognitive wireless system based on available statistical channel state information (CSI) that is applicable to the cognitive system with limited computational resources due to its low complexity. We considered the scenario where the primary and the cognitive network coexist in the same spectrum band, under the constraints of interference threshold and maximal tolerable outage permitted by the primary user. The communication in the secondary decode-and-forward (DF) relaying system is established via a self-sustainable relay, which harvests energy from both cognitive and primary transmitters. The closed-form expressions for the outage probability of the cognitive network are derived, which are valid for both time-switching relaying (TSR) and power-splitting relaying (PSR) protocols. We analyze the influence of both cognitive and primary systems as well as the impact of channel parameters on the cognitive system outage performance. The derived analytical results are corroborated by an independent simulation method.

## 1. Introduction

Recent advances in telecommunication concepts have resulted in ubiquitous connectivity, as well as a variety of possible services of improved quality. However, due to increasing demands for dedicated spectrum resources, it is necessary to have extremely efficient and innovative approaches to the usage of limited spectrum resources. The traditional static spectrum allocation has led to its inefficient use (both in time and at various locations) and consequently to its scarcity for the deployment of new services. It is envisioned that forthcoming networks will satisfy a wide range of requirements regarding different types of services, so spectrum access should be adjusted with the aim to maximize overall spectrum utilization. This is especially important for the Internet of Things (IoT) systems that have an exceptionally important role in transforming the way communications are used in everyday life. IoT systems integrated with 5G offer new services and smart communications such as the smart grid, smart health, smart traffic, etc. Nevertheless, as IoT systems imply a huge number of spatially distributed connected devices and sensors with modest powering and computational capabilities, access to spectrum resources needs to be provided in a manner that is adapted to their characteristics and limitations [1,2].

It is well known that cognitive radio can significantly improve spectrum efficiency by adopting an underlay, overlay, or interweave transmission mode [3]. While the interweave approach involves energy consuming spectrum sensing, the underlay approach enables a coexistence of licensed and unlicensed networks. In the underlay concept, the cognitive, secondary user (SU) communicates concurrently in the same spectrum band dedicated to the existing primary user (PU) without causing harmful interference to the PU. SUs apply the real-time transmit power adaptation based on the available channel state information (CSI), such that the interference level caused at the licensed user’s receiver is within predetermined tolerable limits [4,5]. However, the fulfillment of this condition is possible only in the case when perfect CSI is available to the SU, which is not practically achievable. In the underlay cognitive system, the CSI can be provided to the SU through cooperation with the PU (if the channel is reciprocal) or by mediation of the band-manager. However, the acquired CSI is practically always imperfect, even in the case when there is cooperation with the PU [6,7,8,9,10]. Due to the time-varying nature of the channel and the finite backward channel latency, the acquired CSI is outdated. Even in the case when the propagation environment is slow varying, the backward channel quantization results in an imperfect CSI. In the absence of cooperation, only statistical CSI (i.e., long-term CSI) can be available [10,11,12,13]. Notably, in any case when perfect CSI is not available, the strict interference constraint cannot be satisfied, and the interference threshold will be exceeded during a certain percentage of time, resulting in degraded primary and secondary system performance. The adaptation of transmission power based on real-time updating of the CSI knowledge is not desirable in the case when there are a large number of cognitive nodes with limited computational capabilities. The underlay cognitive network based on statistical CSI enables a less complex solution for coexistence with the primary spectrum user’s network.

Apart from the constant demands for improved spectrum efficiency, the relentlessly increasing number of devices in a network also necessitates energy-efficient solutions. In this paper, we focus on the application of energy-harvesting techniques [14,15,16,17,18,19], which can improve the performance of an underlay cognitive radio system. In the rest of this section, we will provide an overview of related work, summary of the contributions, and explain the organization of the paper. 

### 1.1. Related Work

The performances of underlay cognitive radio with available imperfect CSI are analyzed in [6,7,8]; these works introduce the concept of interference outage, as the strict interference threshold constraint at the PU receiver cannot be satisfied. The underlay cognitive network based on statistical CSI enables lower complexity solution for coexistence with the primary spectrum user’s network [9]. In the paper [10], the outage probability and ergodic capacity were obtained for the case when transmit power adaptation is done based on the CSI that is outdated, as well as for the case when only statistical properties of the channel from the SU transmitter to the PU receiver are known. The impact of CSI knowledge on the underlay system performances is analyzed in the context of relaying [11,12] and application of space-time codes under multiple interferers’ environment [13]. 

The energy-harvesting technology represents a promising way to enable a practically feasible and energy-efficient powering of the nodes [14]. Although energy can be harvested from various natural sources (solar, thermal, vibration energy, etc.) [15], harvesting radio frequency (RF) energy is very appealing, as it provides necessary reliability in the energy supply [16]. In the field of harvesting energy from RF sources, technology has achieved many advances both in industry [17] and academia [18] in recent years, and much more is expected in the future [19].

The performance analysis of the wireless network where a transmitter harvests energy from both interference and ambient RF sources is presented in [20]. The combined use of a cognitive network [21,22] with the cooperative energy-harvesting approach [23,24,25] can further enable the coexistence of two networks in the same spectrum band with the controlled mutual interference that enables energy sustainability of network relay nodes [26].

The concept of cooperative decode-and-forward (DF) relay that harvests energy from both source and interference signals in the Rayleigh fading environment is proposed in [24] and further analyzed in [27] for Nakagami-*m* environment and nonlinear energy harvester. Energy harvesting is analyzed in [28] for cooperative relaying cognitive network with perfect CSI, and in [29,30,31] for the case of imperfect CSI. However, to the best of authors’ knowledge, there is no available investigation concerning the impact of the statistical CSI on the performance of the cognitive cooperative relaying network that employs energy harvesting. 

### 1.2. Summary and Organization

In this paper, we provide a comprehensive outage analysis of the cooperative cognitive system with a DF relaying scheme. The relay is energy-constrained and relies on the energy harvested from both primary and secondary transmitters, which coexist in the same spectrum band by using statistical CSI knowledge. Two important cases are analyzed: harvesting at the relay node is performed based on time-switching relaying (TSR) or power-splitting relaying (PSR) protocols, as proposed in [32]. It is assumed that the communication between nodes in the cooperative cognitive system is dominantly limited by the interference signals generated by the PU’s system. 

We derive closed-form expressions for the outage probability of the cognitive energy-harvesting DF relaying system constrained by the interference threshold at the primary receiver and tolerable outage probability of the primary system. The derived results are valid for the Rayleigh fading environment. It is important to emphasize that the Rayleigh fading model encompasses the path-loss effect, while it does not provide shadowing effects. In the considered system model, we assume that there are no obstacles between nodes that would introduce a shadowing effect. The numerical results are obtained on the basis of derived expressions and corroborated by using an independent simulation method for various system parameters and propagation conditions.

The rest of the paper is organized as follows. System and channel models are described in Section 2, while outage probability analysis is provided in Section 3. Numerical results, based on both analytical and simulation approach, are presented in Section 4. The final conclusions are outlined in Section 5.

## 2. System and Channel Models

**Figure 1:**
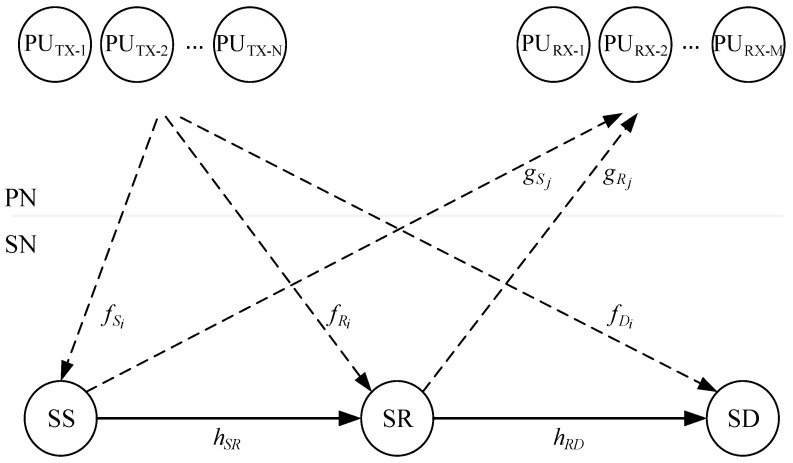
Model of the underlay cognitive relay network.

This paper considers a cognitive cooperative radio system in which secondary users coexist with the primary users of the spectrum in the same frequency band under the predefined conditions regarding permitted introduced interference. The model of the analyzed cognitive relay system is shown in Figure 1. Our further analysis is based on the following set of assumptions:

A1. The secondary system consists of the secondary source (SS), the energy constrained secondary relay node (SR), and the destination of the cognitive secondary system (SD). There is no direct link between the SS and the SD due to the occurrence of deep fading, so the SS and the SD can communicate only with the help of the SR [11,24,32,33,34].

A2. The secondary system shares the spectrum with the primary network based on the underlay paradigm, which means that secondary users can perform concurrent transmission as long as the interference generated at the primary receiver does not exceed a permissible threshold. It is assumed that only statistical CSI is available to the secondary network, i.e., that the mean channel power gain values are known (long-term CSI). The strict interference power constraint cannot be satisfied in this scenario, and the outage interference power constraint is applied as in [10,11,12,13]. 

A3. The primary network consists of *N* transmitters (PU_TX_) and *M* receivers (PU_RX_). PU transmitters are closely positioned at one location, while all PU_RX_ are closely positioned at another location [12,33,34]. This corresponds to the case of cooperative distributed network where the corresponding PU_TX_ form a transmit virtual antenna array [35], or the case where PU’s transmitters and PU’s receivers are grouped in two wireless sensor network node clusters [36]. 

A4. The fading envelopes in all channels follow the Rayleigh distribution. This model encompasses the path loss effect as the mean channel power gain equals $d^{-\beta }$, where *d* denotes the distance between the transmitter and the receiver, while β is the path loss exponent. 

A5. In the interference-limited case, the interference power caused by PU transmitters at the SR and the SD is dominant relative to the noise power [11,12,33,34].

A6. The SR does not have its own power supply, and it harvests energy from the secondary source and the *N* transmitters of the primary network. According to the applied protocol, all the energy harvested during a scheduled frame is used for information transmission [24,32,34]. 

As it is shown in Figure 1, the channel gain coefficients from the SS to the SR and from the SR to the SD are denoted by *h_SR_* and *h_RD_*, respectively. Furthermore, the channel gain coefficients from the SS and the SR to the PU_RX-*j*_ are denoted by $g_{S_{j}}$ and $g_{R_{j}}$ for *j* = 1, 2, …, *M*, respectively. Finally, the channel gain coefficients from the PU_TX-*i*_ to the SS, the SR and the SD are denoted by $f_{S_{i}}$, $f_{R_{i}}$ and $f_{D_{i}}$ for *i* = 1, 2, …, *N*, respectively.

The secondary transmitter is allowed to transmit in the same frequency band under the constraint that the peak interference threshold, denoted by *Q_p_*, is exceeded with a maximum tolerable probability $P_{out,PU}$. Therefore, the maximal transmitted power at the SS, *P_S_*, is determined by the permitted interference outage probability, i.e.,

(1) $$\mathrm{Pr}\left\{P_{S}g_{S_{j}}<Q_{p}\right\}=1-P_{out,PU},$$

where Pr{⋅} denotes probability. 

The transmit power of the SS is adjusted by using the mean value of the power gain in the links from the SS to the PU’s receivers

(2) $$P_{S,\max}=k_{S}\frac{Q_{p}}{\Lambda_{S}},$$

where *k_S_* is the coefficient of adjustment of the SS transmit power and $\Lambda_{S}$ is the mean value of all power gain coefficients ${\left|g_{S_{j}}\right|}^{2}$ (*j* = 1, 2, …, *M*) in the links from the SS to each of the PU’s receivers. 

The transmit power of the SR is adjusted by using the mean value of the power gain in the links from the SR to the PU’s receivers

(3) $$P_{R,max}=k_{R}\frac{Q_{p}}{\Lambda_{R}},$$

where *k_R_* is the coefficient of the adjustment of the SR transmit power and $\Lambda_{R}$ is the mean value of all power gain coefficients ${\left|g_{R_{j}}\right|}^{2}$ (*j* = 1, 2, …, *M*) in the links from the SR to each of the PU’s receivers.

### 2.1. Time-Switching Relaying Protocol for Energy Harvesting

**Figure 2:**
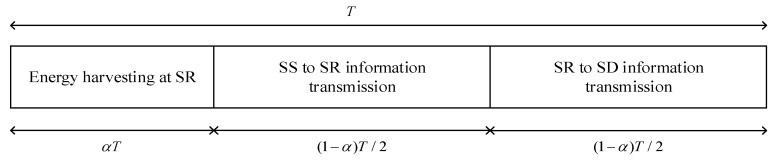
Transmission block structure in the TSR scheme.

Figure 2 depicts the transmission block structure in the TSR scheme for energy harvesting and information processing at the SR. In the figure, *T* represents the time frame interval and *α* ∈ {0, 1} denotes the fraction of the block time in which the SR harvests energy from the RF signals from the SS and *N* PU’s transmitters. The remaining block time (1 − *α*)*T* is used for the information transmission in such a way that half of that time, (1 − *α*)*T*/2, is used for the information transmission from the SS to the SR, while the remaining half, (1 − *α*)*T*/2, is used for the information transmission from the SR to the SD. 

The received baseband signal at the SR is given by

(4) $$y_{R}=\sqrt{P_{S,\max}}h_{SR}s+\sum_{i=1}^{N}\sqrt{P_{PU-TX,i}}f_{R_{i}}s_{PU-TX,i}+n_{R},$$

where *s* is the information signal sent from the SS, *P_PU-TX,i_* is the power of the signal from the i-th PU transmitter, the interference signal from PU*_TX-i_* (with the unit power) is denoted by *s_PU-TX,i_* and the *n_R_* is additive white Gaussian noise component at the relay node.

We consider the case of interference-limited environment, where the interference power caused by *N* PU’s transmitters at the relay node SR is dominant relative to the noise power at the relay and therefore can be neglected in derivations. Then, the received SIR at the SR can be written in the form

(5) $$\gamma_{R}=\frac{P_{S,\max}{\left|h_{SR}\right|}^{2}}{\sum_{i=1}^{N}P_{PU-TX,i}{\left|f_{R_{i}}\right|}^{2}}=\frac{\gamma_{SR}}{I_{R}},$$

where $\gamma_{SR}=P_{S,\max}{\left|h_{SR}\right|}^{2}$ and $I_{R}=\sum_{i=1}^{N}P_{PU-TX,i}{\left|f_{R_{i}}\right|}^{2}$.

The harvested energy at the relay node of the secondary system is

(6) $$E_{H}=\eta \left(P_{S,\max}{\left|h_{SR}\right|}^{2}+\sum_{i=1}^{N}P_{PU-TX,i}{\left|f_{R_{i}}\right|}^{2}\right)\alpha T=\eta \left(\gamma_{SR}+I_{R}\right)\alpha T,$$

where *η* (0 < *η* < 1) is the energy conversion efficiency coefficient that depends on the energy harvester.

It is assumed that the total energy harvested at the SR during the dedicated time can be stored in a storage device (e.g., a supercapacitor or a short-term/high-efficiency battery) and be used afterwards for the power transmission in the same time frame. Therefore, for the harvested energy *E_H_*, the maximal available transmit power of the SR can be expressed as

(7) $$P_{R,EH}^{}=\frac{E_{H}}{\left(1-\alpha \right)T/2}.$$

The transmit power of the energy constrained relay node is determined by both harvested energy and maximal power permitted due to interference outage constraint. Using Equations (3) and (7), the transmit power of the SR can be obtained as

(8) $$P_{R}=\min\left(P_{R,EH},k_{R}\frac{Q_{p}}{\Lambda_{R}}\right).$$

The transmit power of the secondary relay can be further expressed in the following form

(9) $$P_{R}=\left\{ \begin{array}{ll} \frac{2\eta \alpha }{\left(1-\alpha \right)}(\gamma_{SR}+I_{R}), & \frac{2\eta \alpha }{\left(1-\alpha \right)}(\gamma_{SR}+I_{R})\leq k_{R}\frac{Q_{p}}{\Lambda_{R}},\\ k_{R}\frac{Q_{p}}{\Lambda_{R}}, & \frac{2\eta \alpha }{\left(1-\alpha \right)}(\gamma_{SR}+I_{R})>k_{R}\frac{Q_{p}}{\Lambda_{R}}. \end{array}\right.$$

Then, the received information signal at the secondary destination is equal

(10) $$y_{D}=\sqrt{P_{R}}h_{RD}s_{R}+\sum_{i=1}^{N}\sqrt{P_{PU-TX,i}}f_{D_{i}}s_{PU-TX,i}+n_{D},$$

where *s_R_* is the decoded information signal sent from the secondary relay, the *s_PU-TX,i_* is the interference signal from the *i*-th primary user transmitter, and *n_D_* is the additive white Gaussian noise component at the secondary destination. The interference power caused by *N* PU’s transmitters at the SD is dominant relative to the noise power; i.e., the communication is interference-limited. The received SIR at the SD can be written in the form

(11) $$\gamma_{D}=\frac{P_{R}{\left|h_{RD}\right|}^{2}}{\sum_{i=1}^{N}P_{PU-TX,i}{\left|f_{D_{i}}\right|}^{2}}=P_{R}\frac{\gamma_{RD}}{I_{D}},$$

where $\gamma_{RD}={\left|h_{RD}\right|}^{2}$ and $I_{D}=\sum_{i=1}^{N}P_{PU-TX,i}{\left|f_{D_{i}}\right|}^{2}$.

By using Equations (9) and (11), the expression for the received SIR at the SD becomes

(12) $$\gamma_{D}=\left\{ \begin{array}{ll} \frac{2\eta \alpha }{\left(1-\alpha \right)}\frac{\gamma_{RD}}{I_{D}}(\gamma_{SR}+I_{R}), & \frac{2\eta \alpha }{\left(1-\alpha \right)}(\gamma_{SR}+I_{R})\leq k_{R}\frac{Q_{p}}{\Lambda_{R}},\\ k_{R}\frac{Q_{p}}{\Lambda_{R}}\frac{\gamma_{RD}}{I_{D}}, & \frac{2\eta \alpha }{\left(1-\alpha \right)}(\gamma_{SR}+I_{R})>k_{R}\frac{Q_{p}}{\Lambda_{R}}. \end{array}\right.$$

### 2.2. Power-Splitting Relaying Protocol for Energy Harvesting

**Figure 3:**
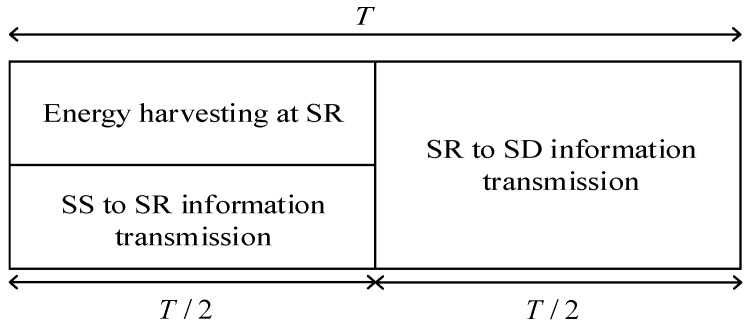
Transmission block structure in the PSR scheme.

The transmission block structure for the information and power transmission according to the PSR protocol is presented in Figure 3. The total time *T* is split into two equal parts. In the first part of duration equal to *T*/2, the source transmits information to the relay, and in the second part of duration equal to *T*/2, the relay further transmits information to the destination. In addition to the information transmission, the first part is used for the energy harvesting. The fraction of the received signal power equal *ρP* (0 < *ρ* < 1) is used for that purpose, while the remaining received power equal (1 − *ρ*)*P* is used for the signal transmission from the source to the relay.

The received baseband signal at the SR is given by

(13) $$y_{R}=\sqrt{\left(1-\rho \right)P_{S,\max}}h_{SR}s+\sum_{i=1}^{N}\sqrt{\left(1-\rho \right)P_{PU-TX,i}}f_{R_{i}}s_{PU-TX,i}+n_{R},$$

where *s* is the information signal sent from the SS, the *s_PU-TX,i_* is the interference signal from the *i*-th primary user transmitter and with the power equal *P_PU-TX,i_*, while *n_R_* is the additive white Gaussian noise component at the SR. As in the paper [24], the case when power splitting reduces the signal power and not the noise power is considered, which provides a lower-bound system performances. 

The interference power caused by *N* primary user transmitters at the SR is dominant relative to the noise component power and can be neglected in the further calculations. The received SIR at the secondary relay node can be written in the following form

(14) $$\gamma_{R}=\frac{\left(1-\rho \right)P_{S,\max}{\left|h_{SR}\right|}^{2}}{\sum_{i=1}^{N}\left(1-\rho \right)P_{PU-TX,i}{\left|f_{R_{i}}\right|}^{2}}=\frac{P_{S,\max}{\left|h_{SR}\right|}^{2}}{\sum_{i=1}^{N}P_{PU-TX,i}{\left|f_{R_{i}}\right|}^{2}}=\frac{\gamma_{SR}}{I_{R}},$$

where $\gamma_{SR}=P_{S,\max}{\left|h_{SR}\right|}^{2}$ and $I_{R}=\sum_{i=1}^{N}P_{PU-TX,i}{\left|f_{R_{i}}\right|}^{2}$.

The energy harvested at the SR is equal to

(15) $$E_{H}=\eta \rho \left(P_{S,\max}{\left|h_{SR}\right|}^{2}+\sum_{i=1}^{N}P_{PU-TX,i}{\left|f_{R_{i}}\right|}^{2}\right)T/2=\eta \rho \left(\gamma_{SR}+I_{R}\right)T/2.$$

Similarly as in the case of TSR scheme, the maximal available transmit power of the SR for the harvested energy *E_H_* can be expressed as

(16) $$P_{R,EH}=\frac{E_{H}}{T/2}.$$

We assume that the SR transmits with the maximal allowed power, which is limited by both the quantity of the harvested energy and the interference constraint imposed by the primary network. By using Equations (3) and (16), the transmit power of SR can be expressed as

(17) $$P_{R}=\min\left(P_{R,EH},k_{R}\frac{Q_{p}}{\Lambda_{R}}\right),$$

and the transmitted power of the secondary relay can be further presented in the following form

(18) $$P_{R}=\left\{ \begin{array}{ll} \eta \rho (\gamma_{SR}+I_{R}), & \eta \rho (\gamma_{SR}+I_{R})\leq k_{R}\frac{Q_{p}}{\Lambda_{R}},\\ k_{R}\frac{Q_{p}}{\Lambda_{R}}, & \eta \rho (\gamma_{SR}+I_{R})>k_{R}\frac{Q_{p}}{\Lambda_{R}}. \end{array}\right.$$

The received information signal at the SD is

(19) $$y_{D}=\sqrt{P_{R}}h_{RD}s_{R}+\sum_{i=1}^{N}\sqrt{P_{PU-TX,i}}f_{D_{i}}s_{PU-TX,i}+n_{D},$$

where *s_R_* is the decoded information signal sent from the *s*_R_, *s_PU-TX,i_* is the interference signal from the *i*-th primary user transmitter, and *n_D_* is the AWGN component at the SD.

The interference power caused by *N* primary user transmitters at the SD is dominant relative to the noise power and the noise impact can be neglected in the further analysis. The received SIR at the SD can be written in the form

(20) $$\gamma_{D}=\frac{P_{R}{\left|h_{RD}\right|}^{2}}{\sum_{i=1}^{N}P_{PU-TX,i}{\left|f_{D_{i}}\right|}^{2}}=P_{R}\frac{\gamma_{RD}}{I_{D}},$$

where $\gamma_{RD}={\left|h_{RD}\right|}^{2}$ and $I_{D}=\sum_{i=1}^{N}P_{PU-TX,i}{\left|f_{D_{i}}\right|}^{2}$.

By using Equations (18) and (20), the expression for the received SIR at the SD becomes

(21) $$\gamma_{D}=\left\{ \begin{array}{ll} \eta \rho \frac{\gamma_{RD}}{I_{D}}(\gamma_{SR}+I_{R}), & \eta \rho (\gamma_{SR}+I_{R})\leq k_{R}\frac{Q_{p}}{\Lambda_{R}},\\ k_{R}\frac{Q_{p}}{\Lambda_{R}}\frac{\gamma_{RD}}{I_{D}}, & \eta \rho (\gamma_{SR}+I_{R})>k_{R}\frac{Q_{p}}{\Lambda_{R}}. \end{array}\right.$$

## 3. Outage Probability Analysis

The outage probability of the secondary network is defined as the probability that one of the links is below predefined threshold $\gamma_{th}$, and it is given by the following expression

(22) $$P_{OUT}(\gamma_{th})=\mathrm{Pr}\left\{\gamma_{R}\leq \gamma_{th}\right\}+\mathrm{Pr}\left\{\gamma_{D}\leq \gamma_{th},\gamma_{R}>\gamma_{th}\right\}$$

It can be noticed that the SIR at the SD for both TSR and PSR protocols, given by Equations (12) and (21) respectively, can be expressed in the unique form and re-written as

(23) $$\gamma_{D}=\left\{ \begin{array}{ll} c\frac{\gamma_{RD}}{I_{D}}(\gamma_{SR}+I_{R}), & c(\gamma_{SR}+I_{R})\leq q,\\ q\frac{\gamma_{RD}}{I_{D}}, & c(\gamma_{SR}+I_{R})>q, \end{array}\right.$$

where $q=k_{R}\frac{Q_{p}}{\Lambda_{R}}$, $c=c^{TS}=\frac{2\eta \alpha }{\left(1-\alpha \right)}$ for the TSR protocol and $c=c^{PS}=\eta \rho$ for the PSR protocol.

By using Equations (22) and (23), the following expression is obtained

(24) $$\begin{array}{ll} P_{OUT}\left(\gamma_{th}\right) & =\mathrm{Pr}\left\{\frac{\gamma_{SR}}{I_{R}}\leq \gamma_{th}\right\}+\mathrm{Pr}\left\{c\frac{\gamma_{RD}}{I_{D}}\left(\gamma_{SR}+I_{R}\right)\leq \gamma_{th},c(\gamma_{SR}+I_{R})\leq q,\frac{\gamma_{SR}}{I_{R}}>\gamma_{th}\right\}\\ & +\mathrm{Pr}\left\{q\frac{\gamma_{RD}}{I_{D}}\leq \gamma_{th},c(\gamma_{SR}+I_{R})>q,\frac{\gamma_{SR}}{I_{R}}>\gamma_{th}\right\}. \end{array}$$

By substituting $u=\gamma_{SR}+I_{R}$, $\nu =\frac{\gamma_{SR}}{I_{R}}$ and $w=\frac{\gamma_{RD}}{I_{D}}$, the outage probability can be written as

(25) $$P_{OUT}\left(\gamma_{th}\right)=\mathrm{Pr}\left\{v\leq \gamma_{th}\right\}+\mathrm{Pr}\left\{u\leq \min\left(\frac{\gamma_{th}}{cw},\frac{q}{c}\right),v>\gamma_{th}\right\}+\mathrm{Pr}\left\{w\leq \frac{\gamma_{th}}{q},u>\frac{q}{c},v>\gamma_{th}\right\},$$

and it can be further written in the form more convenient for the analysis that follows

(26) $$P_{OUT}\left(\gamma_{th}\right)=\mathrm{Pr}\left\{v\leq \gamma_{th}\right\}+\mathrm{Pr}\left\{u\leq \frac{\gamma_{th}}{cw},w>\frac{\gamma_{th}}{q},v>\gamma_{th}\right\}+\mathrm{Pr}\left\{w\leq \frac{\gamma_{th}}{q},v>\gamma_{th}\right\}=I_{1}+I_{2}+I_{3}.$$

The propagation channels among primary and secondary transmitters and the receivers are subject to Rayleigh distributed fading. Therefore, the random variables (RVs) $\gamma_{SR}$ and $\gamma_{RD}$ are Gamma distributed, and the corresponding probability density functions (PDFs) are given by

(27) $$p_{\gamma_{SR}}\left(x\right)=\frac{1}{\Omega_{SR}}\exp\left(-\frac{x}{\Omega_{SR}}\right),$$

(28) $$p_{\gamma_{RD}}\left(x\right)=\frac{1}{\Omega_{RD}}\exp\left(-\frac{x}{\Omega_{RD}}\right),$$

with the mathematical expectations (mean values) equal to $\Omega_{SR}=E\left[P_{S,\max}{\left|h_{SR}\right|}^{2}\right]$ and $\Omega_{RD}=E\left[{\left|h_{RD}\right|}^{2}\right]$, respectively. Furthermore, the sum of *N* independent Gamma distributed variables follows χ^2^-distribution with 2*N* degrees of freedom [37] and

(29) $$p_{I_{R}}\left(x\right)=\frac{x^{N-1}}{\Gamma \left(N\right)\Omega_{I_{R}}^{N}}\exp\left(-\frac{x}{\Omega_{I_{R}}}\right),\;\Omega_{I_{R}}=E\left[P_{PU-TX,i}{\left|f_{R_{i}}\right|}^{2}\right],$$

(30) $$p_{I_{D}}\left(x\right)=\frac{x^{N-1}}{\Gamma \left(N\right)\Omega_{I_{D}}^{N}}\exp\left(-\frac{x}{\Omega_{I_{D}}}\right),\;\Omega_{I_{D}}=E\left[P_{PU-TX,i}{\left|f_{D_{i}}\right|}^{2}\right],$$

where Γ(⋅) denotes Gamma function [38] (8.310.1). 

Further calculations with appropriate mathematical operations presented in Appendix A lead to the final closed-form expression for the outage probability. 

The probability defined by integral *I*_1_ can be calculated in the following way

(31) $$I_{1}=\mathrm{Pr}\left\{\nu \leq \gamma_{th}\right\}=1-\frac{1}{{\left(1+\frac{\Omega_{I_{R}}}{\Omega_{SR}}\gamma_{th}\right)}^{N}}$$

The probability defined by the expression *I_2_* can be further expressed as

(32) $$I_{2}=\mathrm{Pr}\left\{u\leq \frac{\gamma_{th}}{cw},w>\frac{\gamma_{th}}{q},v>\gamma_{th}\right\}=\int_{\frac{\gamma_{th}}{q}}^{\infty }\left(\int_{0}^{\frac{\gamma_{th}}{cw}}\int_{\gamma_{th}}^{\infty }p_{u,v}\left(u,v\right)dvdu\right)p_{w}\left(w\right)dw$$

By mathematical derivations described in Appendix B, Equation (32) is written in the following closed form

(33) $$I_{2}=\Omega_{I_{R}}^{-N}{\left(\frac{1}{\Omega_{I_{R}}}-\frac{1}{\Omega_{SR}}\right)}^{-N}\left(I_{21}-I_{22}-\sum_{k=0}^{N-1}\frac{\left(1+\gamma_{th}\right)\Omega_{I_{R}}}{k!\left(\gamma_{th}\Omega_{I_{R}}+\Omega_{SR}\right)}{\left(\frac{\Omega_{SR}-\Omega_{I_{R}}}{\gamma_{th}\Omega_{I_{R}}+\Omega_{SR}}\right)}^{k}\left(\Gamma \left(1+k\right)I_{21}-I_{23}\right)\right),$$

where integrals *I_21_* and *I*_22_ are given with

(34) $$I_{21}=\int_{\frac{\gamma_{th}}{q}}^{\infty }p_{w}\left(w\right)dw={\left(\frac{\Omega_{I_{D}}}{\Omega_{RD}}\frac{\gamma_{th}}{q}+1\right)}^{-N},$$

and

(35) $$\begin{array}{l} I_{22}=\int_{\frac{\gamma_{th}}{q}}^{\infty }e^{-\frac{\gamma_{th}}{\Omega_{SR}cw}}p_{w}\left(w\right)dw=Ne^{\frac{\gamma_{th}}{c\;\Omega_{SR}}\frac{\Omega_{I_{D}}}{\Omega_{RD}}}\sum_{i=0}^{N-1}\left(\begin{array}{c} N-1\\ i \end{array}\right){\left(-1\right)}^{N-1-i}{\left(\frac{\Omega_{I_{D}}}{\;\Omega_{RD}}\right)}^{N-i}\times \\ \left({\left(\frac{\Omega_{I_{D}}}{\;\Omega_{RD}}\right)}^{i-N}E_{i}\left(1-i+N,\frac{\gamma_{th}}{c\;\Omega_{SR}}\frac{\Omega_{I_{D}}}{\Omega_{RD}}\right)-{\left(\frac{\Omega_{I_{D}}}{\;\Omega_{RD}}+\frac{q}{\gamma_{th}}\right)}^{i-N}E_{i}\left(1-i+N,\left(\frac{\Omega_{I_{D}}}{\;\Omega_{RD}}+\frac{q}{\gamma_{th}}\right)\frac{\gamma_{th}}{c\Omega_{SR}}\right)\right), \end{array}$$

while integral *I*_23_ is defined and solved as

(36) $$\begin{array}{l} I_{23}=\int_{\frac{\gamma_{th}}{q}}^{\infty }\Gamma \left(1+k,\frac{\xi }{w}\right)p_{w}\left(w\right)dw=k!\frac{N\Omega_{I_{D}}}{\Omega_{RD}}\sum_{j=0}^{k}\frac{1}{j\;!}{\left(\xi \right)}^{j}e^{\xi \frac{\Omega_{I_{D}}}{\Omega_{RD}}}\sum_{i=0}^{N+j-1}\left(\begin{array}{c} N+j-1\\ i \end{array}\right){\left(-\frac{\Omega_{I_{D}}}{\Omega_{RD}}\right)}^{N+j-1-i}\\ \times \left({\left(\frac{\Omega_{I_{D}}}{\Omega_{RD}}\right)}^{i-N}E_{i}\left(1-i+N,\frac{\Omega_{I_{D}}}{\Omega_{RD}}\xi \right)-{\left(\frac{\Omega_{I_{D}}}{\Omega_{RD}}+\frac{q}{\gamma_{th}}\right)}^{i-N}E_{i}\left(1-i+N,\left(\frac{\Omega_{I_{D}}}{\Omega_{RD}}+\frac{q}{\gamma_{th}}\right)\xi \right)\right), \end{array}$$

where $\xi =\frac{\gamma_{th}\Omega_{I_{R}}+\Omega_{SR}}{\Omega_{SR}\Omega_{I_{R}}\left(1+\gamma_{th}\right)}\frac{\gamma_{th}}{c}$.

The detailed derivations of integrals *I*_2*i*_, *i* = 1, 2, 3 are provided in Appendix B. 

The probability defined by integral *I*_3_ is equal to

(37) $$\begin{array}{ll} I_{3} & =\mathrm{Pr}\left\{w\leq \frac{\gamma_{th}}{q},v>\gamma_{th}\right\}=\mathrm{Pr}\left\{w\leq \frac{\gamma_{th}}{q}\right\}\mathrm{Pr}\left\{v>\gamma_{\mathrm{th}}\right\}\\ & =\left(1-{\left(1+\frac{\Omega_{I_{D}}}{\Omega_{RD}}\frac{\gamma_{th}}{q}\right)}^{-N}\right){\left(1+\frac{\Omega_{I_{R}}}{\Omega_{SR}}\gamma_{th}\right)}^{-N}. \end{array}$$

## 4. Simulation Method and Numerical Results

In this section, we provide the numerical results for the outage probability and the throughput of the proposed cognitive EH relay system with available statistical CSI. The results are presented for various system parameters and propagation scenarios. The numerical results are obtained by applying the derived analytical closed-form expressions and verified by an independent simulation method. Simulation results are obtained by using a Monte Carlo method, based on waveform sequences with *L* = 10^7^ samples. As it will be shown in the following part, the results obtained by both approaches are in excellent agreement.

### 4.1. Simulation Environment

The simulation environment is developed to verify the accuracy of the derived analytical expressions. It is based on Monte Carlo simulations, which is typically used to estimate performance of the wireless communication systems.

The channel gains are represented as mutually independent random processes with Rayleigh distribution. The mean channel gains from the group of primary users in the same cluster to the other node have the same value, i.e., the corresponding random variables are independent but identically distributed (i.i.d.). Waveform sequences are generated for all channel coefficients by using an improved Jakes fading simulator [39], with *M*_0_ = 20 oscillators and maximum Doppler frequency *f*_D,max_ = 20 Hz (this value does not affect the simulation results, as temporal fading characteristics are not relevant in this system scenario; if the outdated CSI was analyzed instead of statistical SCI, it would be significant). Therefore, sequences *h_SR_*(*k*), *h_RD_*(*k*), *g_S__j_*(*k*), *g_R__j_*(*k*), *f_R__i_*(*k*), and *f_D__i_*(*k*) are generated for *i* = 1, 2, …, *N*, *j* = 1, 2, …, *M*, and *k* = 1, 2, …, *L* where *L* is the number of samples in each waveform and every sample corresponds to one time slot. 

The system simulation is done based on the available statistical CSI, meaning that the transmitted power of the secondary user is adapted based on mean channel power gain values. Therefore, the maximum output power at the SS is determined by using Equation (2), where the coefficient of adjustment is calculated from (1) for given *P*_out,PU_ and *Q_p_*. Depending on the used protocol, further analysis differs, which is shown by using different equations for calculations. In both protocols, as described in Section 2, the SR harvests energy from the SS and from *N* transmitters of the primary network. As parameters *P*_PU-TX-I_, *P*_S,max_, α, η, and *T* are fixed and known and the waveforms *h_SR_*(*k*) and *f_R_*(*k*) are generated, the harvested energy in the *k*-th slot at the relay node of the secondary system is calculated using Equations (6) and (15) for TSR and PSR protocol, respectively. Depending on the harvested energy and maximal allowable power, the transmit power of the SR in the *k*-th slot is adjusted by using the mean value of the power gain in the channels from the SR to the PU’s receivers. It is obtained using Equations (8) and (17) for TSR and PSR protocol, respectively.

We consider the case of interference-limited environment, where the interference power caused by *N* PU’s transmitters at the SR or SD is dominant relative to the corresponding noise power. The signal-to-interference ratio on the SR in the *k*-th slot is obtained using Equations (5) and (14) for TSR and PSR protocol, respectively. In addition, the signal-to-interference ratio at the SD in *k*-th slot is obtained using Equations (11) and (20) for TSR and PSR protocol, respectively. The outage probability of the secondary system is estimated by comparing values of the signal-to-interference ratio at the SR and the SD nodes with the outage threshold in each particular slot. 

### 4.2. Numerical Results

In this section, we present numerical results for outage probability and achievable throughput for both analyzed protocols. Numerical results that correspond to the analytical approach are obtained directly from Equations (26) and (31)–(37). We also wrote the scripts for estimating outage probability for a proposed cognitive system by using independent Monte Carlo simulation method, as described in previous subsection. The results are presented for various values of parameters *Q_p_*, *P_out,PU_*, and for a few values of mean channel power gains.

**Figure 4:**
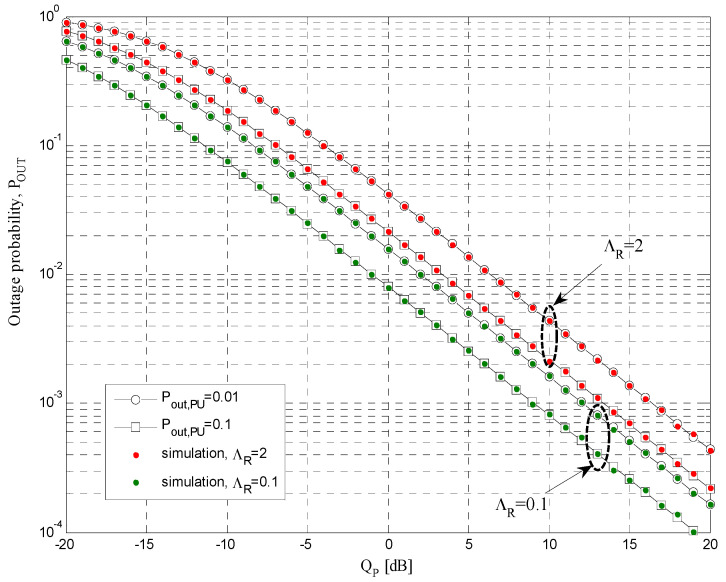
Outage probability *P_OUT_* vs. *Q_p_* for TSR protocol.

**Figure 5:**
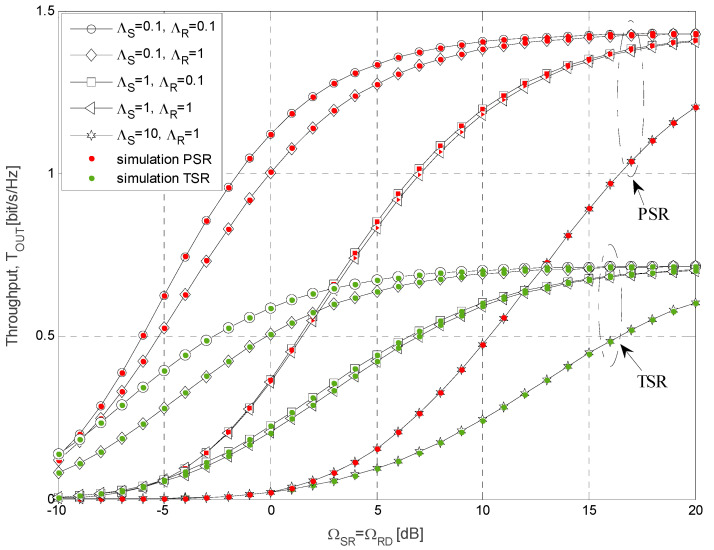
Throughput for different values of mean channel power gain in a cognitive relay system.

**Figure 6:**
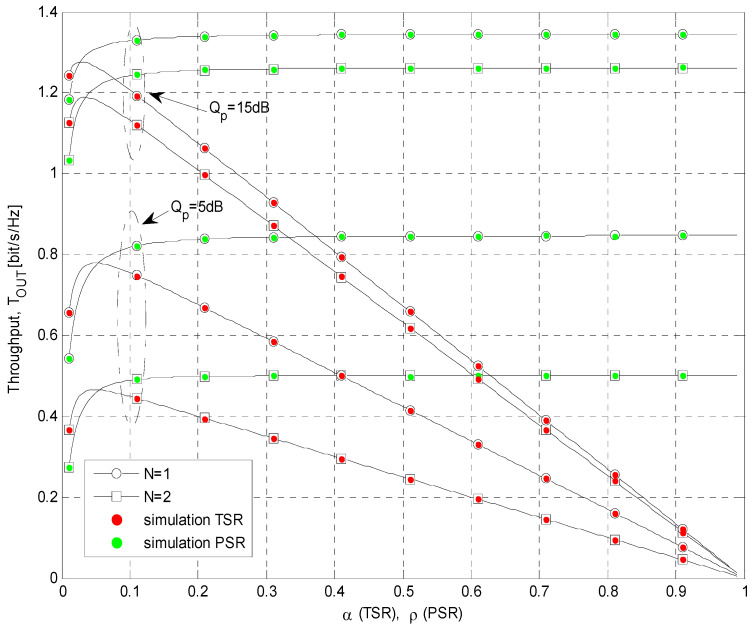
Throughput vs. energy harvesting ratio.

**Figure 7:**
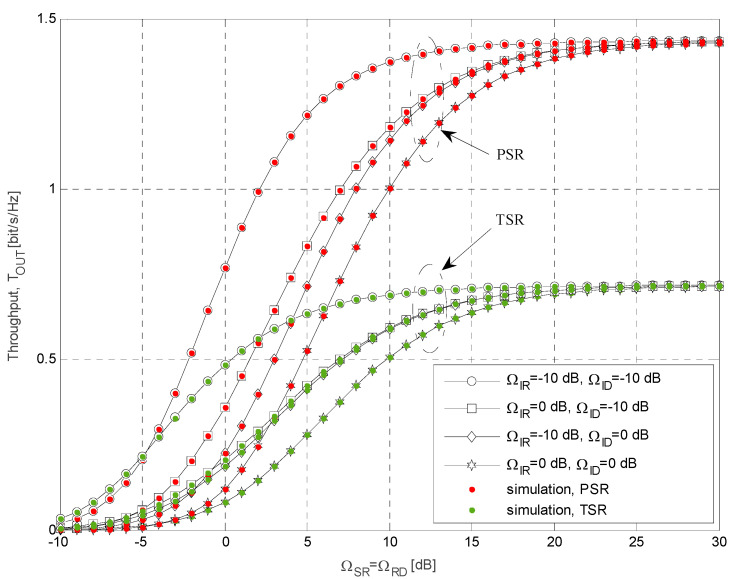
The impact of the interference power at the secondary source and the relay on the throughput values for various Ω*_SR_* = Ω*_RD_*.

**Figure 8:**
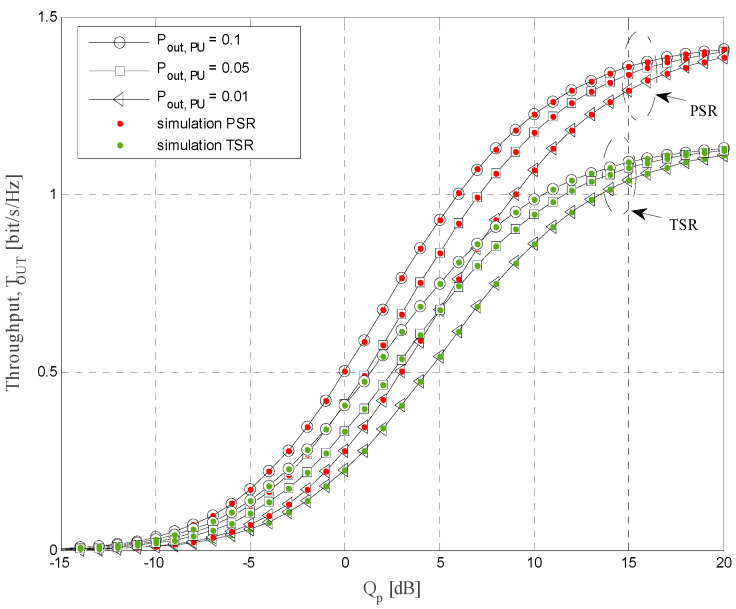
The achievable throughput vs. interference threshold *Q_p_*.

**Figure 9:**
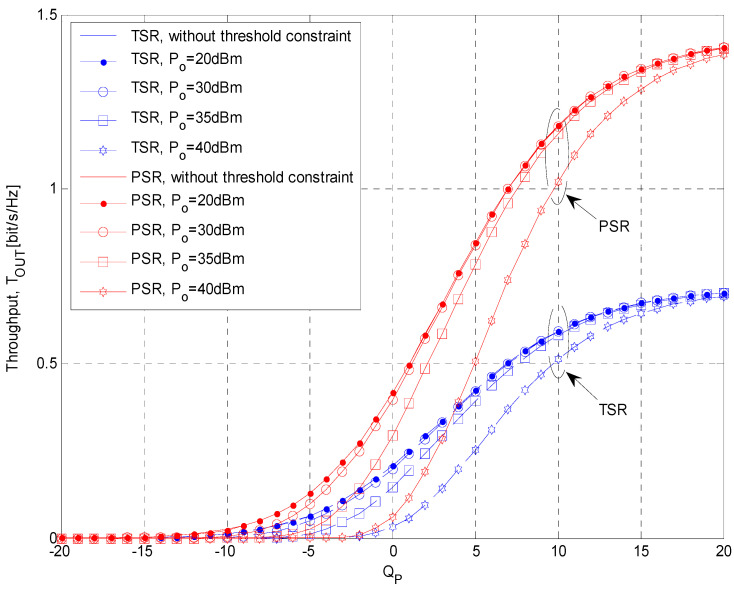
Simulation results for the achievable throughput vs. interference threshold *Q_p_* and minimum threshold constraints of the input power at the SR, *P_o_*.

The simulation parameters that are used for calculations of the results presented in Figure 4, Figure 5, Figure 6, Figure 7, Figure 8 and Figure 9 are shown in Table 1. In all simulations, the energy conversion efficiency coefficient is *η =* 0.9. The number of primary transmitters is set to *N* = 1 in all simulations, except in the case of the results presented in Figure 6, where it is considered that the number of primary transmitters is *N* = 2. The simulation results are obtained based on waveform sequences with *L* = 10^7^ samples. As the estimated outage probability values for various input parameters are not lower than 10^−4^ for any set of system and channel parameters, the obtained estimations can be considered accurate with high confidence level [40]. 

The dependence of outage probability on the interference threshold *Q_p_* is examined, and the results are presented in Figure 4 for the case of TSR protocol. Scenarios with different values of primary network outage probability *P_out,PU_* and mean channel power gain Λ*_R_* from the secondary relay to the primary receiver are considered. The mean channel power gain Λ*_S_* from the secondary source to the primary receiver is set to 0.01, the energy harvesting ratio is 0.5, the mean channel power gains are Ω*_I__R_* = 0 dB, Ω*_I__D_* = −10 dB, Ω*_SR_* = 10 dB, and Ω*_RD_* = 10 dB, while the primary user transmitters have the unit power and the outage threshold is *γ_th_* = −5 dB. By increasing the interference threshold *Q_p_,* the outage probability decreases. This result can be explained by fact that the increase of the interference threshold *Q_p_* leads to higher permitted transmit power values at the secondary source, as well as more harvested energy and higher permitted power at the relay.

The outage probability values decrease for smaller values of $\Lambda_{R}$, as the allowed transmit power of the secondary relay is higher. Moreover, for higher values of permitted primary network outage probability *P_out,PU_*, the outage probability of the cognitive network is lower. The reason for this effect can be explained by the fact that with the higher maximal allowed value of outage probability (for the fixed interference threshold *Q_p_*), both the transmit power of the secondary source and the allowed power at the relay are increasing, and the received signal-to-interference ratios at both the secondary relay and the destination are higher. Similar results are obtained for PSR protocol using the same scenarios.

In the next figures, the achievable throughput of the energy-harvesting DF system with statistical CSI is analyzed. The achievable throughput depends on the applied protocol, so for TSR and PSR protocol, it can be expressed, respectively, as [24,41]

(38) $$T_{OUT}^{TSR}=(1-\alpha )C_{OUT},$$

(39) $$T_{OUT}^{PSR}=C_{OUT},$$

where *C_OUT_* is outage capacity, which is defined as a maximum data rate that can be achieved in the channel with the outage probability $P_{OUT}(\gamma_{th})=\mathrm{Pr}(\gamma <\gamma_{th})$

(40) $$C_{OUT}=0.5(1-P_{OUT}(\gamma_{th}))log_{2}(1+\gamma_{th}).$$

More specifically, in the DF system, outage probability P*_OUT_* is defined with Equation (22).

In Figure 5, the throughput is presented as a function of mean channel power gains Ω*_SR_* and Ω*_RD_* for both analyzed energy-harvesting protocols, where the outage probability of the primary link is set to *P_out,PU_* = 0.05 and the permitted interference threshold is *Q_p_* = 10 dB. The impact of different values of the mean channel power gain $\Lambda_{S}$ from the secondary source to the primary receivers and the mean channel power gain $\Lambda_{R}$ from the secondary relay to the primary receivers is considered. The energy conversion efficiency coefficient is *η* = 0.9, while the time-splitting factor and power-splitting factor are equal to 0.5 for TSR and PSR protocols, respectively. The mean channel power gains in the interference channels from the primary transmitters to secondary relay and destination are Ω*_IR_* = 0 dB and Ω*_ID_* =−10 dB, respectively. We analyzed the scenario where mean values of channel power gain in both source-to-relay and relay-to-destination links are equal and the outage threshold is *γ_th_* = 8 dB. The obtained results demonstrate that in all considered cases, the throughput values increase with the raise of mean channel power gains Ω*_SR_ =* Ω*_RD_* when these values are below 10 dB, while the saturation effect occurs for the higher values of mean channel power gain.

Furthermore, the saturation occurs for higher Ω*_SR_ =* Ω*_RD_* when the mean channel power gain $\Lambda_{S}$ is higher, as the transmit power of the secondary relay is then dominantly determined by the interference constraint of the primary user. This can be explained by the fact that in this case, the maximal allowed transmit power of the secondary source is smaller, and the interference constraint at the relay becomes dominant only for higher mean channel power gain Ω*_SR_*. In accordance to the expectations, in all analyzed cases, greater throughput values are achieved in the case when PSR energy harvesting protocol is applied. It can be noticed that the throughput increases with the decrease of mean channel power gain $\Lambda_{S}$, as in that case, the higher secondary source transmit power is permitted. Similarly, the throughput values increase for smaller values of $\Lambda_{R}$, as in that case, the allowed transmit power of the secondary relay is higher. However, in the case when $\Lambda_{S}=10$, the difference between cases of various $\Lambda_{R}$ values is diminishing, as outage performances are dominantly limited by the source-to-relay link. 

The dependence of the cognitive system throughput on the time-splitting factor *α* and power-splitting factor *ρ* is presented in Figure 6, for different values of primary user’s interference threshold *Q_p_* and number of primary transmitters *N*. The obtainable throughput is analyzed for both TSR and PSR protocols, with the energy conversion efficiency coefficient *η* = 0.9. The maximal permitted probability of the primary link outage is set to *P_out,PU_* = 0.05, the outage threshold of the cognitive system is *γ_th_* = 8 dB. In this scenario, the mean channel power gains are equal to Ω*_IR_* = 0 dB, Ω*_ID_* = −10 dB, Ω*_SR_* = 10 dB, and Ω*_RD_* = 10 dB. Moreover, in the case when TSR protocol is applied, one can identify the optimal value of time-splitting factor *α* when the throughput is maximized. This value is achieved for values of *α* < 0.05, while a higher increase of time-splitting factor leads to the throughput decrease. On the other hand, in the case of energy-harvesting PSR protocol, the throughput increases with the power-splitting factor for small values of *ρ*, as then, more power is dedicated to energy harvesting, and the transmit power of the relay is increasing. However, in the case of further increase, the saturation occurs, as the maximal relay transmit power is determined with the more strict restriction imposed by the primary network and the average SIR at the relay is independent of *ρ*. 

Furthermore, better system performances are achieved with a smaller number of transmitters in the primary network for both energy-harvesting protocols. This can be explained by the fact that although the interference from the primary network is used for energy harvesting, the degrading effect of the interference is more dominant. Finally, from the obtained results, it can be concluded that the throughput always increases with the raise of the allowed interference threshold *Q_p_*. A bigger value of *Q_p_* increases both the total input power *P_S_* as well as the permitted transmit power at the relay, therefore resulting in better system throughput.

In Figure 7, the throughput is shown as the function of Ω*_SR_* and Ω*_RD_* for both analyzed energy harvesting protocols. We considered scenarios with different values of mean channel power gains Ω*_IR_* and Ω*_ID_* in the interference channels from the primary transmitter to both relay and destination nodes, respectively. The outage probability of primary link is set to 0.05 and the interference threshold is *Q_p_* = 10 dB (the time-switching and power-splitting factors are *α* = *ρ* = 0.5 and the outage threshold is *γ_th_* = 8 dB). The mean value of the mean channel power gain from both the secondary source and relay to the primary receivers are $\Lambda_{S}=\Lambda_{R}=1$. The best system performances are achieved for the smallest values of Ω*_ID_* and Ω*_IR,_* for both analyzed protocols. Furthermore, the increase of Ω*_IR_* and Ω*_ID_* has a negative influence on the system performances. In the case of PSR protocol, obtained results are better when Ω*_IR_* = 0 dB and Ω*_ID_ =* −10 dB than when Ω*_IR_* = −10 dB, Ω*_ID_ =* 0 dB, while in the case of TSR protocol, the difference is smaller, and these curves are almost overlapping. This effect can be explained by the fact that the interference at the destination has only a degrading effect, whereas at the relay interference, it also increases harvested energy. The throughput increases with the raise of Ω*_SR_ =* Ω*_RD_,* and in the region of high values, it is the same for all analyzed values of Ω*_IR_* and Ω*_ID_*. That is because the transmit power of the energy-constrained relay node is determined by the harvested energy and the maximal power permitted due to the interference outage constraint. That effect is noticeable also in the case of PSR protocol for values of Ω*_IR_* and Ω*_ID_* higher than 25 dB.

Throughput values are presented in Figure 8 as a function of the permitted interference threshold *Q_p_* for both energy-harvesting protocols (*α* = *ρ* = 0.2) and different values of primary network outage probability. The mean channel power gains are Ω*_IR_* = 0 dB, Ω*_ID_* = −10 dB, Ω*_SR_* = 10 dB, Ω*_RD_* = 10 dB, and the threshold is *γ_th_* = 8 dB. For low values of *Q_p_*, throughput values are very low, for both protocols and all analyzed values of primary network outage probability. For small values of *Q_p_*, the total transmit power is very low, the amount of harvested energy is limited, and the transmit power of the secondary relay is consequently low. By increasing the permitted interference threshold *Q_p_*, values of the system throughput are rising, as more energy is harvested and the obtainable transmit power of the secondary relay is higher. Moreover, the permitted values of the secondary relay transmit power is increasing for higher *Q_p_* as the interference constraint is relaxed. For higher values of *Q_p_*, better system performances are obtained with applied PSR protocol (than TSR). Moreover, better performances are achieved for higher values of permitted primary network outage probability *P_out,PU_*. The reason for this effect can be explained by the fact that with higher maximal allowed value of outage probability (for the fixed interference threshold *Q_p_*), both the transmit power of the secondary source and the allowed power at the relay are increasing, and the received signal-to-interference ratios at the secondary relay and the destination are higher.

Although the used system model is widely accepted and utilized in various scenarios of cognitive and energy-harvesting systems, it has some weaknesses and limitations. As explained in assumption A3, our analysis is limited to the case when all PU transmitters are grouped in one cluster, and all PU receivers are grouped in the other cluster (as a special case, it is valid for the case of single PU_TX_ and single PU_RX_). In addition, we assumed in A4 that the line-of-sight propagation component is not present and the shadowing effect can be neglected, although this assumption is not reasonable in some propagation scenarios. More importantly, the energy-harvesting model is an idealization of the realistic process. 

The realistic energy-harvesting model has the minimum amount of energy necessary for the harvesting process, as SR consumes energy in the process of energy harvesting [42]. Therefore, we will obtain simulation results for different values of minimum threshold constraints of the input power at the SR node, *P_o_*, which is needed for achieving energy harvesting gain.

Throughput values estimated by using Monte Carlo simulation method are presented in Figure 9, as a function of the permitted interference threshold *Q_p_* for both energy-harvesting protocols (*α* = *ρ* = 0.5) and different values of *P_o_*. The outage threshold is *γ_th_* = 8 dB, while the mean channel power gain values are set to Ω*_IR_* = 0 dB, Ω*_ID_* = −10 dB, Ω*_SR_* =10 dB, and Ω*_RD_* =10 dB. For low values of *Q_p_*, throughput values are very low, for both protocols and all analyzed values of *P_o_*. This can be explained by the fact that for small values of *Q_p_*, the total input power at the SR is very low, so it is difficult to reach a defined minimum threshold of input power needed for achieving energy-harvesting gain. By increasing the permitted interference threshold *Q_p_*, the total input power at the SR is also increasing, more energy is harvested, and the obtainable transmit power of the SR is higher, resulting in the improved system throughput values. Moreover, the permitted value of the SR transmit power is increasing for higher *Q_p_* as the interference constraint is relaxed. For higher values of *Q_p_*, better system performances are obtained with applied PSR protocol (than TSR). 

It is obvious that better performances are achieved for lower values of minimum threshold *P_o_*, because the probability that the total input power of SR exceeds these values is higher. In the case when the total input power of the SR is below *P_o_*, there is no energy harvesting gain at the SR, and the output power of the SR is equal to zero. For values of minimum threshold constraints of the SR input power equal to 20 dBm, system performances are comparable with the ones obtained in the case when there is no threshold constraints (i.e., *P_o_* = 0 W). It is important to highlight that all considered values are significantly higher than values previously used in the literature (P_o_ = −12 dBm or 64 μW in [42]).

## 5. Conclusions

In this paper, we analyzed a cognitive DF relaying system with multiple primary transmitters and primary receivers. The self-sustainable relay harvests energy from both cognitive and primary transmitters based on TSR and PSR energy-harvesting protocols. The transmission among nodes in the cognitive relaying network is constrained by the maximal tolerable outage probability and the interference threshold permitted by the primary user. The closed-form expressions for outage probability and achievable throughput have been derived for the interference-limited case and the Rayleigh propagation environment. All derived analytical results are confirmed by an independent simulation method. 

It has been shown that a higher value of the interference threshold increases the permitted transmit power at the SS, enabling a larger amount of harvested energy and higher obtainable transmit power of the SR. As the increased interference threshold value also results in higher allowable transmit power at the SR (for the given amount of harvested energy), the resulting outage probability decreases, and system throughput improves. The performances of the proposed system are also improved for higher permitted primary network outage probability. The throughput values of the system increase with the raise of the mean channel power gains from the SS to the SR and from the SR to the SD when these values are below a certain value, while the saturation effect occurs for the higher values of the mean channel power gain. Furthermore, the decreased values of the mean channel power gain from the SS to the PU’s receivers and from the SR to the PU’s receivers result in better system performances. The opposite happens with the increase of the mean channel power gains from the PU’s transmitters to the SR and from the PU’s transmitters to the SD. In the case when TSR protocol is applied, one can identify the optimal value of the time-splitting factor *α* when the throughput is maximized. After that value, a further increase of the time-splitting factor leads to the throughput decrease. On the other hand, in the case of the energy-harvesting PSR protocol, the throughput increases with the power-splitting factor for small values of *ρ*, while further increase leads to saturation.

Finally, the provided analysis and derived closed-form expressions open up new research topics for further investigations. Due to limited computational capabilities of the proposed network and limited energy of the SR, it is a challenging task to provide secure communication in the secondary network. In such a scenario, physical layer security has some benefits when compared to complex cryptography techniques that ensure a high level of security. Therefore, the first task in the future work will be to determine the secrecy capacity of the secondary link in the scenario analyzed in this paper. Furthermore, the presented analysis can be extended to the case when the propagation environment is modeled with Nakagami-*m* distribution. This is a generalization of the considered scenario; as for the case when the fading parameter is *m* = 1, it corresponds to the analysis of the Rayleigh fading environment presented in this paper. Furthermore, as the analyzed statistical channel model encompasses the path-loss effect, the impact of network geometry and distances among nodes can be investigated based on the results provided in the paper. This type of analysis can offer some valuable guidelines for the optimal design of the cognitive energy-harvesting relaying system that maximizes both primary and secondary system performances. However, the considered model does not include the shadowing effect, so the analysis can be extended to the case when shadowing is also modeled (using log-normal or generalized Gamma fading distribution). Future work can also analyze some specific scenarios where channel power gains from the same group of users differ due to a non-uniform distribution of small-sized obstacles in the environment. 

## Figures and Tables

**Table 1:** Simulation parameters.

Parameter	Figure 4	Figure 5	Figure 6	Figure 7	Figure 8	Figure 9
*P_out,PU_*	0.01–0.1	0.05	0.05	0.05	0.01–0.1	0.05
*Q_p_* [dB]	−20–20	10	5–15	10	−15–20	−15–20
$\Lambda_{R}$	0.1–2	0.1–1	1	1	1	1
$\Lambda_{S}$	0.01	0.1–10	1	1	1	1
Ω*_I__R_* [dB]	0	0	0	−10–0	0	0
Ω*_I__D_* [dB]	−10	−10	−10	−10–0	−10	−10
Ω*_SR_* [dB]	10	−10–20	10	−10–30	10	10
Ω*_RD_* [dB]	10	−10–20	10	−10–30	10	10
*γ_th_* [dB]	−5	8	8	8	8	8
*η*	0.9	0.9	0.9	0.9	0.9	0.9
*α*	0.5	0.5	0.01–0.99	0.5	0.2	0.5
*ρ*	0.5	0.5	0.01–0.99	0.5	0.2	0.5
*N*	1	1	1–2	1	1	1

## Appendix A

In this part, we derive the mathematical statistics for random variables *v* i *w*.

As random variable is defined as $\nu =\gamma_{SR}/I_{R}$, using [43] (7.23), we obtain the corresponding PDF as

(A1) $$p_{v}\left(v\right)=\int_{0}^{\infty }xp_{\gamma_{{}_{SR}}}\left(vx\right)p_{I_{R}}\left(x\right)dx.$$

Furthermore, by substituting corresponding PDF expressions, the following PDF is obtained

(A2) $$p_{\nu }\left(\nu \right)=\frac{N\Omega_{I_{R}}}{\Omega_{SR}}\frac{1}{{\left(\frac{\Omega_{I_{R}}\nu }{\Omega_{SR}}+1\right)}^{N+1}}$$

On the basis of the previous expression, the CDF expression for the random variable *v* is obtained, and it is given by Equation (31).

Following the same approach, we obtain the PDF and the CDF expressions for the random variable $w=\gamma_{RD}/I_{D}$ and they are given, respectively, as

(A3) $$p_{w}\left(w\right)=\frac{N\Omega_{I_{D}}}{\Omega_{RD}}\frac{1}{{\left(\frac{\Omega_{I_{D}}w}{\Omega_{RD}}+1\right)}^{N+1}},$$

(A4) $$\mathrm{Pr}\left\{w\leq \gamma_{th}\right\}=1-\frac{1}{{\left(1+\frac{\Omega_{I_{D}}}{\Omega_{RD}}\gamma_{th}\right)}^{N}}$$

## Appendix B

To solve the integral *I_2_*, the joint PDF for $u=\left(\gamma_{\mathrm{SR}}+I_{R}\right)$ and $v=\gamma_{\mathrm{SR}}/I_{R}$ is needed. With the help of $p_{u,v}\left(u,v\right)=\left|J\right|p_{\gamma_{\mathrm{SR}}}\left(\frac{uv}{v+1}\right)p_{I_{D}}\left(\frac{u}{v+1}\right)$ [43] (pp. 7–37), where $\left|J\right|=\frac{u}{{\left(v+1\right)}^{2}}$ is Jacobian determinant, and replacing appropriate PDFs, the joint PDF is found as

(A5) $$p_{u,v}\left(u,v\right)=\frac{1}{\Gamma \left(N\right)\Omega_{I_{R}}^{N}\Omega_{SR}}\frac{u^{N}}{{\left(v+1\right)}^{N+1}}\exp\left(-\frac{1}{\Omega_{SR}}\frac{uv}{v+1}\right)\exp\left(-\frac{1}{\Omega_{I_{R}}}\frac{u}{v+1}\right).$$

We first solve integral over *ν*, $\int_{\gamma_{th}}^{\infty }p_{u,v}\left(u,v\right)dv$, by using the substitution $t={\left(v+1\right)}^{-1}$ and by using [37] (3.351.2) and following expression is obtained

(A6) $$\int_{\gamma_{th}}^{\infty }p_{u,v}\left(u,v\right)dv=\frac{{\left(\frac{1}{\Omega_{I_{R}}}-\frac{1}{\Omega_{SR}}\right)}^{-N}}{\Gamma \left(N\right)\Omega_{I_{R}}^{N}\Omega_{SR}}\exp\left(-\frac{u}{\Omega_{SR}}\right)\left(\Gamma \left(N\right)-\Gamma \left(N,\left(\frac{1}{\Omega_{I_{R}}}-\frac{1}{\Omega_{SR}}\right)\frac{u}{1+\gamma_{th}}\right)\right),$$

where Γ(⋅,⋅) is the incomplete Gamma function [38] (8.350.2).

Further, integration over *u* is performed by using serial representation of incomplete Gamma function [37] (3.351.2) and applying [38] (3.351.1)

(A7) $$\begin{array}{l} \int_{0}^{\frac{\gamma_{th}}{cw}}\int_{\gamma_{th}}^{\infty }p_{u,v}\left(u,v\right)dvdu=\frac{1}{\Omega_{I_{R}}^{N}{\left(\frac{1}{\Omega_{I_{R}}}-\frac{1}{\Omega_{SR}}\right)}^{N}}(1-e^{-\frac{\gamma_{th}}{\Omega_{SR}cw}}\\ -\sum_{k=0}^{N-1}\frac{\left(1+\gamma_{th}\right)\Omega_{I_{R}}}{k!\left(\gamma_{th}\Omega_{I_{R}}+\Omega_{SR}\right)}{\left(\frac{\Omega_{SR}-\Omega_{I_{R}}}{\gamma_{th}\Omega_{I_{R}}+\Omega_{SR}}\right)}^{k}\left(\Gamma \left(1+k\right)-\Gamma \left(1+k,\frac{\gamma_{th}\Omega_{I_{R}}+\Omega_{SR}}{\Omega_{SR}\Omega_{I_{R}}\left(1+\gamma_{th}\right)}\frac{\gamma_{th}}{cw}\right)\right)). \end{array}$$

The last, the integral *I*_2_ is given by

(A8) $$\begin{array}{ll} I_{2} & =\frac{1}{\Omega_{I_{R}}^{N}{\left(\frac{1}{\Omega_{I_{R}}}-\frac{1}{\Omega_{SR}}\right)}^{N}}\times \int_{\frac{\gamma_{th}}{q}}^{\infty }(1-e^{-\frac{\gamma_{th}}{\Omega_{SR}cw}}-\sum_{k=0}^{N-1}\frac{\left(1+\gamma_{th}\right)\Omega_{I_{R}}}{k!\left(\gamma_{th}\Omega_{I_{R}}+\Omega_{SR}\right)}{\left(\frac{\Omega_{SR}-\Omega_{I_{R}}}{\gamma_{th}\Omega_{I_{R}}+\Omega_{SR}}\right)}^{k}\\ & \times \left(\Gamma \left(1+k\right)-\Gamma \left(1+k,\frac{\gamma_{th}\Omega_{I_{R}}+\Omega_{SR}}{\Omega_{SR}\Omega_{I_{R}}\left(1+\gamma_{th}\right)}\frac{\gamma_{th}}{cw}\right)\right))p_{w}\left(w\right)dw. \end{array}$$

Integral *I*_2_ can be written in the form as in Equation (33), where expressions *I*_2 *k*_, *k* = 1, 2, 3 are given as

(A9) $$I_{21}=\frac{N\Omega_{I_{D}}}{\Omega_{RD}}\int_{\frac{\gamma_{th}}{q}}^{\infty }{\left(\frac{\Omega_{I_{D}}w}{\Omega_{RD}}+1\right)}^{-N-1}dw={\left(\frac{\Omega_{I_{D}}}{\Omega_{RD}}\frac{\gamma_{th}}{q}+1\right)}^{-N},$$

(A10) $$\begin{array}{l} I_{22}=\int_{\frac{\gamma_{th}}{q}}^{\infty }e^{-\frac{\gamma_{th}}{\Omega_{SR}cw}}p_{w}\left(w\right)dw=\frac{N\Omega_{I_{D}}}{\Omega_{RD}}\int_{\frac{\gamma_{th}}{q}}^{\infty }e^{-\frac{\gamma_{th}}{\Omega_{SR}cw}}{\left(\frac{\Omega_{I_{D}}w}{\Omega_{RD}}+1\right)}^{-N-1}dw=\\ =\frac{N\Omega_{I_{D}}}{\Omega_{RD}}e^{\frac{\gamma_{th}}{c\;\Omega_{SR}}\frac{\Omega_{I_{D}}}{\Omega_{RD}}}\sum_{i=0}^{N-1}\left(\begin{array}{c} N-1\\ i \end{array}\right){\left(-1\right)}^{N-1-i}{\left(\frac{\Omega_{I_{D}}}{\;\Omega_{RD}}\right)}^{N-1-i}\\ \times \left({\left(\frac{\Omega_{I_{D}}}{\;\Omega_{RD}}\right)}^{i-N}E_{i}\left(1-i+N,\frac{\gamma_{th}}{c\;\Omega_{SR}}\frac{\Omega_{I_{D}}}{\Omega_{RD}}\right)-{\left(\frac{\Omega_{I_{D}}}{\;\Omega_{RD}}+\frac{q}{\gamma_{th}}\right)}^{i-N}E_{i}\left(1-i+N,\left(\frac{\Omega_{I_{D}}}{\;\Omega_{RD}}+\frac{q}{\gamma_{th}}\right)\frac{\gamma_{th}}{c\Omega_{SR}}\right)\right), \end{array}$$

(A11) $$\begin{array}{l} I_{23}=\int_{\frac{\gamma_{th}}{q}}^{\infty }\Gamma \left(1+k,\frac{\xi }{w}\right)p_{w}\left(w\right)dw\\ =k!\frac{N\Omega_{I_{D}}}{\Omega_{RD}}\sum_{j=0}^{k}\frac{1}{j\;!}{\left(\xi \right)}^{j}e^{\xi \frac{\Omega_{I_{D}}}{\Omega_{RD}}}\sum_{i=0}^{N+j-1}\left(\begin{array}{c} N+j-1\\ i \end{array}\right){\left(-\frac{\Omega_{I_{D}}}{\Omega_{RD}}\right)}^{N+j-1-i}\\ \times \left({\left(\frac{\Omega_{I_{D}}}{\Omega_{RD}}\right)}^{i-N}E_{i}\left(1-i+N,\frac{\Omega_{I_{D}}}{\Omega_{RD}}\xi \right)-{\left(\frac{\Omega_{I_{D}}}{\Omega_{RD}}+\frac{q}{\gamma_{th}}\right)}^{i-N}E_{i}\left(1-i+N,\left(\frac{\Omega_{I_{D}}}{\Omega_{RD}}+\frac{q}{\gamma_{th}}\right)\xi \right)\right), \end{array}$$

where $\xi =\frac{\gamma_{th}\Omega_{I_{R}}+\Omega_{SR}}{\Omega_{SR}\Omega_{I_{R}}\left(1+\gamma_{th}\right)}\frac{\gamma_{th}}{c}$.
